# Magnetic Resonance Imaging Biomarkers of Muscle

**DOI:** 10.3390/tomography10090106

**Published:** 2024-09-02

**Authors:** Usha Sinha, Shantanu Sinha

**Affiliations:** 1 Department of Physics, San Diego State University, San Diego, CA 92182, USA; 2 Muscle Imaging and Modeling Lab., Department of Radiology, University of California at San Diego, San Diego, CA 92037, USA

**Keywords:** quantitative muscle MRI, fat fraction, T2 mapping, diffusion tensor imaging, neuromuscular disease biomarker

## Abstract

This review is focused on the current status of quantitative MRI (qMRI) of skeletal muscle. The first section covers the techniques of qMRI in muscle with the focus on each quantitative parameter, the corresponding imaging sequence, discussion of the relation of the measured parameter to underlying physiology/pathophysiology, the image processing and analysis approaches, and studies on normal subjects. We cover the more established parametric mapping from T1-weighted imaging for morphometrics including image segmentation, proton density fat fraction, T2 mapping, and diffusion tensor imaging to emerging qMRI features such as magnetization transfer including ultralow TE imaging for macromolecular fraction, and strain mapping. The second section is a summary of current clinical applications of qMRI of muscle; the intent is to demonstrate the utility of qMRI in different disease states of the muscle rather than a complete comprehensive survey.

## 1. Introduction

Quantitative MRI (qMRI) differs from conventional MRI in its ability to provide objective quantitative metrics of the underlying tissue [1]. Conventional MRI is a map of the signal intensities in different tissues where the signal intensities are a complicated function of the acquisition pulse sequence and the underlying tissue parameters. qMRI can capture non-visual metrics related to underlying tissue properties including the chemical structure and biological microstructure. Measurement of each metric, also referred to as parametric mapping (images of the parameter are computed on a voxel basis), requires a specially designed imaging pulse sequence coupled to an underlying biophysical model that provides the link between the observed parameter and underlying tissue properties. qMRI yields numerical values with a unit (e.g., distance, volume, relaxation times) or as a percent (e.g., proton density fat fraction). The qMRI approach provides the basis for developing imaging biomarkers; the latter can have significant clinical impact on diagnostics including earlier detection of disease, in the assessment of disease severity and therapeutic response, and on accurate prognosis. It has the potential to replace or complement biopsies enabling non-invasive assessment of the disease, increasing patient comfort while introducing minimal to no disturbance to the pathology of interest [1]. However, despite the compelling advantages of qMRI based biomarkers, it has yet to be adopted widely in the clinical setting [2]. The slow adoption to clinical practice is due to several reasons: (i) longer duration protocols arising from the need for performing different acquisitions for multi-parametric mapping and (ii) the lack of standards for qMRI including the large number of parametric image acquisition protocols as well as post-processing techniques [3]. The National Institute of Standards and Technology (NIST) hosted workshops in 2014 and 2017 with participants from over 16 organizations working towards standards in quantitative MRI [3]. The recommendations of the workshops included a call for efforts directed at standardizing the imaging and analysis protocols, as well as on developing phantoms with material composition and shape/size appropriate to the particular anatomy and method [3]. The clinical realization of qMRI is anticipated to occur once the standardized acquisition and analysis protocols along with reference phantoms are transferred to the clinic [3]. Toward this, the Radiological Society of North America (RSNA) organized the Quantitative Imaging BioMarkers Alliance (QIBA) [4]. QIBA’s mission is “*to improve the value and practicality of quantitative imaging biomarkers by reducing variability across devices, sites, patients and time*” [4]. Of the 22 imaging biomarker committees that span different modalities, there are 9 committees on qMRI techniques including a qMRI group devoted to musculoskeletal (qMRI-MSK) and another group to quantifying proton density-weighted fat fraction (QIBA-MRI-PDFF) [4].

qMRI has been implemented extensively in the brain characterizing a wide range of neurological diseases, including conditions with inflammatory, cerebrovascular, and neurodegenerative pathologies [5]. A number of recent studies have explored qMRI in oncological applications to characterize malignancies in breast, lung, prostate, and brain cancer, and to either monitor or for early prediction of response to anti-cancer therapies [6]. In the area of musculoskeletal applications of qMRI, there is a Musculoskeletal Biomarkers Committee of the QIBA with a focus on cartilage compositional and morphological characterization [7]. MRI-based cartilage compositional analysis is clinically significant as parametric changes can be identified in the early phases of osteoarthritis before morphological changes are seen in structural MRI [7]. Spin-spin relaxation time (T2) and spin-lattice relaxation time in the rotating frame (T1ρ) have emerged as the most viable approaches for characterizing cartilage composition; T2 reflects changes in water, collagen content, and orientation of collagen fibers, whereas T1ρ is more sensitive to proteoglycan content [7]. 

There are also several studies exploring qMRI in skeletal muscle and several metrics have been extracted and explored for their sensitivity to biochemical and microstructural changes in tissue in normal and in diseased states [8,9,10,11]. It has been applied to characterizing muscle in normal subjects including exploring differences based on age and sex, trained vs. untrained, and the effect of exercise [8]. The utility of qMRI in characterizing several disease conditions including dystrophy, late onset POMPE, and sarcopenia has been reported [12,13,14].

This review is devoted to the current status of quantitative MRI (qMRI) of skeletal muscle. The first section covers the techniques of qMRI in muscle with the focus on each quantitative parameter, the corresponding imaging sequence, discussion of the relation of the measured parameter to underlying physiology/pathophysiology, the image processing and analysis approaches, and studies on normal subjects. We cover the more established parametric mapping from T1-weighted imaging for morphometrics including image segmentation, proton density fat fraction, T2 mapping, and diffusion tensor imaging to more exploratory/less applied qMRI features such as magnetization transfer including ultralow TE imaging for macromolecular fraction and MR strain mapping. The second section is a summary of current clinical applications of qMRI of muscle; the intent is to provide the user a flavor for qMRI in different disease states of the muscle rather than a complete comprehensive survey. We have not included spectroscopy in this review. We refer the interested reader to recent references of experts’ consensus recommendations for proton and for phosphorous magnetic resonance spectroscopy [15,16].

In this review, we present a comprehensive overview of qMRI-based muscle biomarkers including well known (volumetrics, T2, fat quantification, diffusion tensor imaging) and a few emerging metrics (fiber architecture and diffusion data modeling, dynamic strain and strain rate imaging, magnetization transfer contrast and ultrashort echo time imaging). The discussion of the latter emerging techniques as potential imaging-based biomarkers of normal and pathological muscle is not available in the literature. Furthermore, by presenting the application of these techniques in normal muscle and a few select pathologies where qMRI has been more extensively applied, we anticipate that the larger community of researchers and physicians will benefit as they identify potential for qMRI in their areas of muscle pathology. 

## 2. Quantitative Magnetic Resonance Imaging

### 2.1. Muscle Morphology

Muscle volume, anatomical cross-sectional areas (CSA), and physiological cross-sectional areas (PCSA) are predictors of muscle strength [17]. Furthermore, these morphological measures are clinically important in characterizing and tracking the progression of many diseases including muscular dystrophies, myopathies, and sarcopenia [18,19]. While earlier studies extracted fiber lengths from ultrasound to compute PCSA, fiber lengths can now be conveniently determined by combining muscle volume from MR morphological imaging with fiber length from MR diffusion tensor imaging [20]. MR imaging has been successfully used to accurately quantify skeletal muscle volume and cross-sectional area (CSA) with <5% intra- and inter-observer reproducibility in several muscles including thigh and lower leg muscles [21]. However, quantification of muscle volume requires segmentation of the muscle over a stack of slices which is a tedious and time-consuming task if performed manually. In order to obtain a metric that is more readily extracted, Bamman et al. have shown that it is possible to substitute the volume metric by cross-sectional area measurements [17]. Lanza et al. also compared muscle volume to anatomical cross-sectional area metrics and established that the different assessments do not affect the muscle size–strength relationship [22]. 

The T1-weighted fast spin-echo (FSE) sequence is optimal for morphological imaging in terms of image quality and due to its reduced sensitivity to magnetic field inhomogeneities and has thus been used extensively in earlier studies for extracting muscle volume and/or CSA [8]. However, volume acquisitions with gradient echo fat-water Dixon sequences provide speed, excellent SNR, and contrast for segmenting muscles [23]. Susceptibility artifacts are minimized in these sequences by acquiring at low TEs. Another advantage is that the Dixon methods also provide a fat fraction map which can be subtracted from the muscle volume (or CSA) to obtain the muscle volume (or CSA) of contractile tissue corrected for fat [10].

#### Image Processing (Segmentation)

It should be noted that segmentation still remains largely manual in most clinical settings [21]. Manual segmentation cannot be performed readily for 3D datasets for many muscles given that the process is both time intensive and operator dependent [21]. However, widespread use of qMRI for biomarkers depends critically on the ability to segment muscle volumes in an automated fashion [21]. An excellent recent review of the segmentation approaches developed for muscle tissue type and for muscle segmentation is given in Ref. [24].

**Figure 1:**
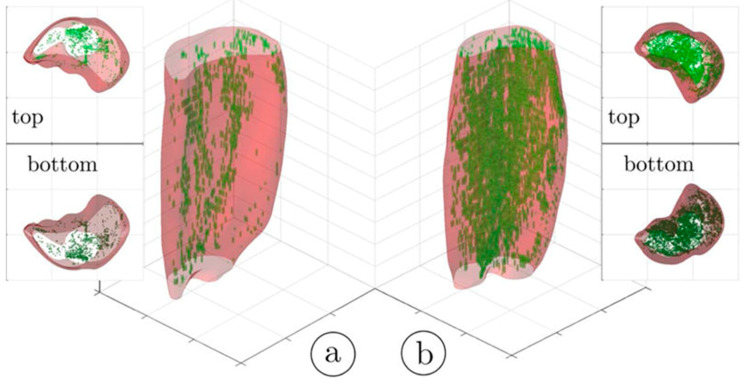
Three-dimensional rendering of the hard thresholded (52%) volumes of IMCT/IMAT tissue in the triceps surae muscles; young subject (**a**) and older subject (**b**). It should be noted that the aponeurosis surrounding each muscle was selectively eroded in order to provide a better view of the IMCT/IMAT. The top and bottom views are given as 3D volume projections. Reproduced with permission from the authors in Ref. [25].

An important segmentation task in muscle diseases is the identification of tissue composition (muscle, adipose, and connective tissue). Fuzzy c-means (FCM) clustering has been proposed that considers partial volume effects at muscle/fat boundaries as well as intramuscular fat infiltration; an extension of the FCM used dual-echo images from an Ultralow TE acquisition to segment adipose and connective tissue from muscle tissue in calf muscle [25]. Figure 1 shows the results of the non-contractile vs. contractile tissue volumes in a young (23-year) and a senior (83-year) participant; the higher amount of adipose and connective (non-contractile) tissue in the senior subject is clearly visualized. 

The identification of the different compartments of fat has clinical implications and includes subcutaneous adipose tissue (SAT) which is separated from the internal adipose tissue (IAT) by fascia (e.g., fascia lata for the thigh). The fat regions within the fascia are further classified into intramuscular fat within each muscle region as well as perimuscular fat between the muscles. To identify the separate deposits of fat, it is important to identify SAT from IAT; the methods to achieve this are detailed in Reference [24]. Various methods have been proposed to identify the inner border of the SAT based on active contours and extensions such as gradient vector flow snakes [26]. Most of these algorithms perform accurately for healthy subjects but not as well in patients with high fat infiltration [24]. The next step is the identification of individual muscle volumes—this is important as fat infiltration occurs differently across muscles and furthermore, there could also be a proximo-distal pattern of infiltration [24]. Segmentation of individual muscles is challenging as muscles have similar intensity and the boundaries between different muscles are thin and often not seen due to partial volume effects. Segmentation strategies based on pre-labeled atlases (manual labeling) use non-linear registration of the new data to the labeled atlas to identify individual muscles. Most atlas-based approaches have been developed for thigh and hip muscles [27] and extending to other muscles would involve developing new atlases and furthermore, the applicability of atlases to identify muscles with high fat filtration has not yet been established. Semi-automated segmentation methods show promise; Ogier et al. used a semi-quantitative method incorporating shape information and non-linear registration to propagate the contour from prior slices for fat quantification in the thighs and lower legs of healthy subjects and patients with myotonic dystrophy type 1 and obtained very good agreement with manual segmentations [28].

Deep Neural Networks (DNNs) have recently made a huge impact in image segmentation including in medical image segmentation [24]. For image segmentation applications, DNNs are supervised learning systems trained on manually segmented images. One of the requirements for robust segmentation is that the DNN be trained with a large number of images. A recent report compared several deep convolutional neural network (CNN) architectures (U-Net 2D, U-Net 3D, TransUNet, and HRNet) for segmenting ten thigh and calf muscles from control and subjects with neuromuscular disease (NMD) [29]. All CNNs performed well with high geometric accuracy for healthy subjects as well as those with NMD; however, the HRNet correctly identified all muscles. A recent publication using DNNs for segmentation focus on lower extremities established that these systems are not only applicable for control healthy subjects but also subjects with pathology [30]. Agosti et al. used MRI data from controls and subjects with facioscapulohumeral dystrophy (FSHD) and amyotrophic lateral sclerosis (ALS) to train CNNs with multi-echo spin echo and a multi-echo gradient echo [31]. The proposed network accurately segmented thigh and calf muscles even in the presence high fat infiltration. Furthermore, the authors have released the automatic segmentation tool resulting as an open-source repository, available at the link in Reference [32].

Deep neural networks hold promise for automated muscle segmentation even in the presence of a high percentage of fat infiltration. While still in a relatively initial phase of development for muscle segmentation, it continues to be an area of intense research activity. It should be noted that most of the semi-automated and automated segmentations have been performed and evaluated on healthy populations and there is a critical need to extend these approaches to large cohorts of subjects with pathological conditions. The applicability of the systems to images from different protocols, scanners, and institutions has to be demonstrated before widespread adoption. The availability of the systems as an open-source repository will clearly be a great start for a larger group of researchers to train and test the DNN systems.

### 2.2. Quantification of Fatty Infiltration in Muscle

Myosteatosis refers to fatty infiltration of skeletal muscle which occurs in a variety of conditions or combination of conditions including aging, disuse, injury, diabetes, and neuromuscular disease [11]. Myosteatosis is associated with loss of muscle mass and strength and increased mortality among the elderly [33]. Adipose tissue is considered an endocrine organ that influences numerous physiological and pathological processes. During weight gain and with aging, adipocytes can reach their capacity to store fat, which increases ectopic storage of fat around and within the non-adipose tissue organs, such as skeletal muscle, liver, and pancreas. In the past decade, myosteatosis has emerged as an important fat depot associated with insulin resistance and Type 2 Diabetes [34]. There are two fat depots within skeletal muscle: fat infiltration within myocytes (intramyocellular fat) and visible fat within the fascia surrounding skeletal muscle (intermuscular fat) [11]. The quantification of both skeletal muscle fat depots can be determined by using invasive analyses such as skeletal muscle biopsy samples, or, by using noninvasive radiological techniques such as computed tomography (CT), magnetic resonance imaging (MRI), and magnetic resonance spectroscopy (MRS).

#### 2.2.1. Fat Quantification Based on Chemical-Shift Encoded (CSE) Imaging

The original two-point Dixon method relies on the differences in resonant frequencies between water and the main fat peak to generate images where fat and water signal are in-phase or out-of-phase at specific echo times [35]. These in- and out-phase images are then combined to yield a magnitude-based fat fraction image. However, the simple two-point Dixon method is prone to errors in fat quantification that arise from various sources: including main magnetic field inhomogeneities, and other confounding factors such as T1, T2, noise bias, T2* correction, spectral complexity of fat, eddy currents, and J-coupling [35]. While the extended Dixon methods address main magnetic field inhomogeneities, they do not address the multiple spectral peaks of fat which leads to fat and water being incompletely separated [35]. The state-of-the-art sequences offer correction for inhomogeneities and confounders and are based on multi-echo SPGR volume acquisitions that model the multiple resonant frequencies of fat, estimate and correct for T2* in the presence of fat, and use a special reconstruction called Iterative Decomposition of Water and Fat with Echo Asymmetry and Least Squares Estimation (IDEAL) to extract the MR-Proton density-weighted fat fraction (MR-PDFF) [36]. This fat quantification sequence or a close variant is available on commercial scanners (IDEAL-IQ on GE, qDIXON on Siemens and mDIXONQuant on Philips scanners) [37]. The QIBA MRI-PDFF committee reported a large-scale study using a commercial PDFF phantom (12 vials with fat fraction from 0 to 100%) that confirmed the accuracy of MRI in determining fat fraction obtained for multiple vendors, at both 1.5 T and 3.0 T, and for multiple pulse sequences [38]. The results from this study provide a measure of confidence to physicians who are using or planning to integrate PDFF as a biomarker for skeletal muscle disease. Given the clinical importance of fat infiltration in skeletal muscle, a number of studies have investigated fat quantification in skeletal muscle. These studies are detailed later in clinical applications of quantitative biomarkers for pathological muscle while studies on normal subjects are summarized here. In this review, we cover pathologies that have been studied fairly extensively by MRI. However, it should be noted that there are several other conditions that can be characterized by qMRI. For example, fatty degeneration of the autochthonous spinal musculature has been found to be associated with fractures of the lower thoracic spine. MRI quantification of fatty infiltration can be a useful biomarker for prediction of fracture risk [39]. PDFF of the paraspinal musculature in normal subjects has been shown to be significantly lower in men compared to women and furthermore, significantly correlated with age [40]. Significant age-related differences in calf muscle composition (adipose from PDFF and fibrosis from ultra-low TE imaging) have been reported in a cross-sectional study of young and senior subjects [41]. In another study, PDFF measurements correlated significantly with paraspinal isometric strength and were a better predictor of paraspinal muscle strength beyond CSA [42]. 

#### 2.2.2. Analysis of Fat Fraction Maps

**Figure 2:**
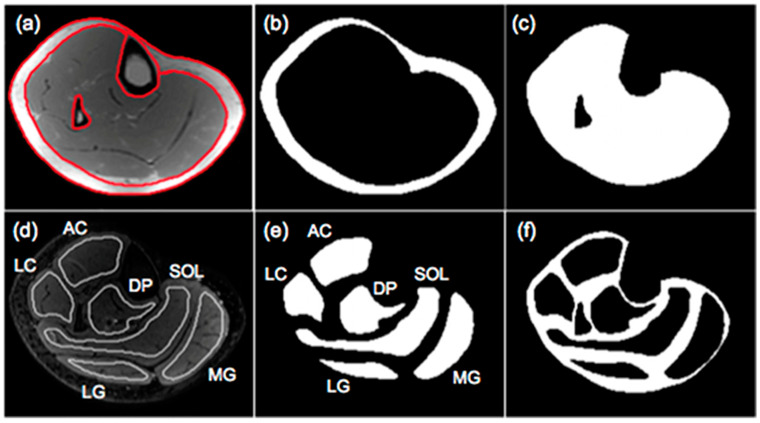
Segmentation of muscle and fat compartments: (**a**) typical in-phase IDEAL image and superimposed ROIs for subcutaneous fat and bone and bone marrow regions, (**b**) subcutaneous adipose tissue (SAT) mask, (**c**) mask including all the muscle regions and excluding the bone and bone marrow regions in the tibia and fibula, (**d**) typical T2-weighted FSE image and superimposed muscular ROIs used for the evaluation of fat distribution, (**e**) masks of 6 muscular ROIs, and (**f**) mask of soft tissue excluding subcutaneous fat and 6 muscular ROIs. Three muscles (medial gastrocnemius-MG, lateral gastrocnemius-LG, soleus-SOL) and three muscle compartments (anterior compartment-AC, lateral compartment-LC, deep posterior compartment-DP) were used to define muscular regions. Fat within the mask of (**c**) corresponds to IMAT, fat within the mask of (**e**) corresponds to intraMF, and the fat within the mask of (**f**) corresponds to interMF. Reproduced with permission from the authors in Ref. [43].

The output of the IDEAL and its variants are separated fat and separated water signal images. In-phase images are then calculated by taking the sum of the separated water and fat images while out-of-phase images are calculated by taking the absolute value of the difference of the separated water and fat images. Fat fraction images are generated from the ratio of the separated fat signal over the sum of the separated water and fat signals [11]. There are several different approaches to analyzing the fat fraction maps. A threshold can be applied to the fat fractions map to identify fat-dominant regions; however, the selection of the threshold is arbitrary and measured adipose tissue volumes will vary with the threshold. Karampinos et al. reported PDFF for calf muscles in subjects with diabetes and provided a comprehensive assessment of PDFF in different compartments including the subcutaneous adipose tissue (SAT), individual muscle ROIs (defined as intramuscle fat, intraMF), and intermuscular fat (denoted as interMF) which is the region between individual muscles (Figure 2) [43]. It should be noted that in the latter work, the segmentations of the individual muscles were eroded to exclude edge pixels (the latter are included in the interMF). The sum of intraMF and interMF is the intramuscular adipose tissue (IMAT). This latter study showed that significant differences between normal and Type 2 Diabetes Mellitus subjects were seen only in interMF and not in IMAT [43]; this finding emphasizes the importance of determining fat fraction separately in different compartments. A note of caution is the decreased ability to obtain accurate fat fraction in the presence of significant fibrosis (e.g., in Duchenne Muscular Dystrophy). The low signal in voxels with fibrotic tissue in both water and fat images can bias the estimation of PDFF.

### 2.3. T2 Mapping

The spin-lattice relaxation time, T2, is sensitive to water mobility in tissue, and since water mobility is very different in the intracellular and extracellular regions, it is reflective of the relative amounts of water in the intracellular and extracellular muscle compartments. In its simplest form, T2 can be measured by the signal decay in two images acquired at two TEs. It is routinely measured by a multi-spin echo (MSE) sequence with a single excitation RF pulse followed by multiple refocusing 180° pulses to acquire images at different TEs; the typical number of acquired echoes is ~15–18 echoes with the first echo acquired at TE time of ~8 ms (min TE) and an echo spacing of ~8 ms [10]. 

Earlier studies on ex vivo tissue identified multi-exponential T2 decay which modeled the decay as arising from multiple compartments [10]. However, Saab et al. showed, using a novel technique with the first echo acquired at TE of 0.6 ms and 2000 echoes that acquired data in a single large voxel, that multi-exponential decay is also present in in vivo muscle tissue [44]. They compared this latter technique with a standard imaging sequence with six echoes and min TE of 18 ms. The former technique revealed multi-exponential relaxation with the lowest T2 component (<5 ms) arising from the hydration shell of macromolecules such as proteins while the longest T2 at 283 ms was a very small fraction and was potentially assigned to either ‘free water’ or vascular blood. Of interest are the three intermediate peaks, the longest T2 component (~100 ms) of these corresponds to water in the interstitial (extracellular) compartment (10% fraction) while the T2 components in the range of 25–45 ms were of intracellular origin (85% fraction). However, the standard imaging sequence yielded 31 ms when the data were fit to a mono-exponential decay. It is important to understand that from a clinical perspective, an increase in the interstitial space and consequently, the extracellular water will result in an increase in the measured T2. It should be noted that elevated T2 values occur in a variety of tissue conditions: inflammation, tumor, necrosis, and denervation, and also in response to the acute changes that occur after exercise of moderate to high intensity. However, though changes in T2 are non-specific, it can still be clinically effective as a tool for monitoring ‘disease activity’, i.e., as a sensitive indicator of disease severity that shows immediate responsiveness to underlying pathological processes [10].

The above background provides the basis for understanding the relationship of T2 to pathology. Skeletal muscle edema can be caused by a number of pathologies including from trauma, early myositis ossificans, and inflammatory myopathies. Edema results in an increase in the interstitial space which results in the increase in T2. This T2 elevation is seen with many pathological conditions (e.g., idiopathic inflammatory myopathies [45], and Duchenne Muscular Dystrophy [12]). It should be noted that the earliest studies employed T2 mapping in order to localize muscle activation as well as to identify patterns of muscle activation with exercise [46]. 

Another aspect of muscle T2 imaging is the infiltration of fat that occurs with age and in most pathological muscle conditions [10]. Since T2 of fat is longer than that of muscle, increases in fat infiltration will lead to elevated T2 values [47]. Fat-suppressed sequences can be used to extract the T2 of muscle [45] but these can suffer from inadequate fat suppression. A different approach taken in other studies is to use the T2 values as a metric of fat infiltration [48]. But this precludes the identification of other intrinsic changes in muscle like inflammation that can also result in T2 elevation. The T2 of water and the T2 of fat can be extracted from a multiexponential fit to the experimental data enabling one to disambiguate the effects of inflammation from that of fat infiltration [8].

There has been a long-standing research effort at implementing T2 mapping that demonstrates spatial patterns and intensity of muscle activation; this approach has also been called muscle functional imaging [46]. It has been well established that muscle T2 increases with exercise [49]. While earlier studies hypothesized that the exercise-induced T2 increases were primarily from an increase in extracellular fluid volume, it is now accepted that the increase in T2 arises from an increase in muscle volume as a consequence of an accumulation of intracellular water driven by osmotically and/or hydrostatically driven fluid shifts [46]. 

#### T2 Analysis

The experimental data can be fit to mono- or multi-exponential fits using nonlinear curve-fitting methods. In order to avoid making assumptions about the number of exponential decays that are required to model the experimental data, nonnegative least squares (NNLS) fitting can be used where the algorithm produces a spectrum of T2 values [10]. When the data are fit to a mono-exponential decay, it yields an average T2 from both the water and fat compartments of muscle and is referred to as the ‘global T2 relaxation time’ [46]. As discussed above, the global T2 will be influenced significantly by the extent of fat infiltration and will shift to longer T2s with higher intramuscular fat. The bias to longer T2s can be understood by comparing the T2 of muscle (33 ms @3T) to that of fat (150 ms @3T); as fat fractions increase (e.g., in patients with muscular dystrophy), ‘global T2 values’ will shift to longer T2s. In order to identify the intrinsic T2 changes in water (muscle) with pathology, one can selectively excite water or suppress fat. Another approach is to fit the data to bi-exponential or tri-exponential fit where the unknowns of the fit are the fat fraction, the T2s of water and fat. This approach requires a fairly large number of echoes for a robust fit as there are many fit parameters. Azzabou et al. reported that a tri-exponential fit to multi-echo data with a 17 echo multi-echo spin-echo sequence [50]. This latter study extracted muscle water T2 which was independent of fat over a large range of fat fraction in muscle. Recently, an Open-Source toolkit for water T2 mapping that implements fast reconstruction enabled by extended phase graphs (EPG) simulations and dictionary matching implemented on a general-purpose graphic processing unit has been reported [51], further enabling T2 mapping to be implemented by the clinical community.

### 2.4. Diffusion Tensor Imaging (DTI)

Diffusion arises from random motion of particles suspended in a liquid or gas and results in a displacement of particles and the square of the average displacement, <*x*^2^>, is governed by Einstein’s diffusion equation, which in 1D is given by <*x*^2^> = 2Dt, where *D* is the diffusion coefficient that quantifies the extent of diffusion and is characteristic of a given tissue and *t* is the diffusion time [52]. The diffusion coefficient in tissue differs from bulk diffusion coefficient in water as the former is hindered by a number of factors including macromolecules and restricted by cells, membrane walls, and permeability. Diffusion in tissue is described by the apparent diffusion coefficient (ADC) to distinguish it from the bulk free diffusion coefficient. 

The measurement of ADC in a diffusion-weighted MRI sequence is accomplished by the addition of strong magnetic field gradients that sensitize the signal to the small displacements arising from diffusion [53]. However, this simultaneously sensitizes the image to physiological and other gross motions that would cause severe artifacts in conventional diffusion-weighted spin-echo sequence. To circumvent these artifacts, a single-shot acquisition called echo planar imaging (EPI) is used which acquires all the data with a single excitation RF pulse [54]. However, this ultrafast technique suffers from low SNR, as well as eddy current, and susceptibility-related artifacts; these latter two effects result in geometric mis-mapping and local deformations as well as signal loss/signal bunching [55]. Post-processing pipelines usually employ different algorithms to denoise as well as to correct for artifacts prior to extraction of diffusion metrics [55]. 

Muscle is a highly organized tissue in which connective tissues (endo-, peri-, and epimysium) create a complex network to enclose fibers, fascicles, and total muscles leading to human skeletal muscles being anisotropic media. An extension of diffusion-weighted imaging is diffusion tensor imaging (DTI) in which diffusion gradients are applied in different directions to extract direction-dependent diffusion [56]. Thus, DTI is ideally suited to explore the anisotropic tissue microstructure as in muscle. The tensor computation process yields the largest diffusion value also denoted as the primary eigenvalue and two smaller diffusion values in two orthogonal directions that are rankedby magnitude as the secondary and tertiary diffusion eigenvalues [56]. Other diffusion metrics include the mean diffusivity (MD) which is the average of the diffusion eigenvalues while the anisotropy of diffusion is captured by the fractional anisotropy (FA) metric (a measure of the difference in eigenvalues).

**Figure 3:**
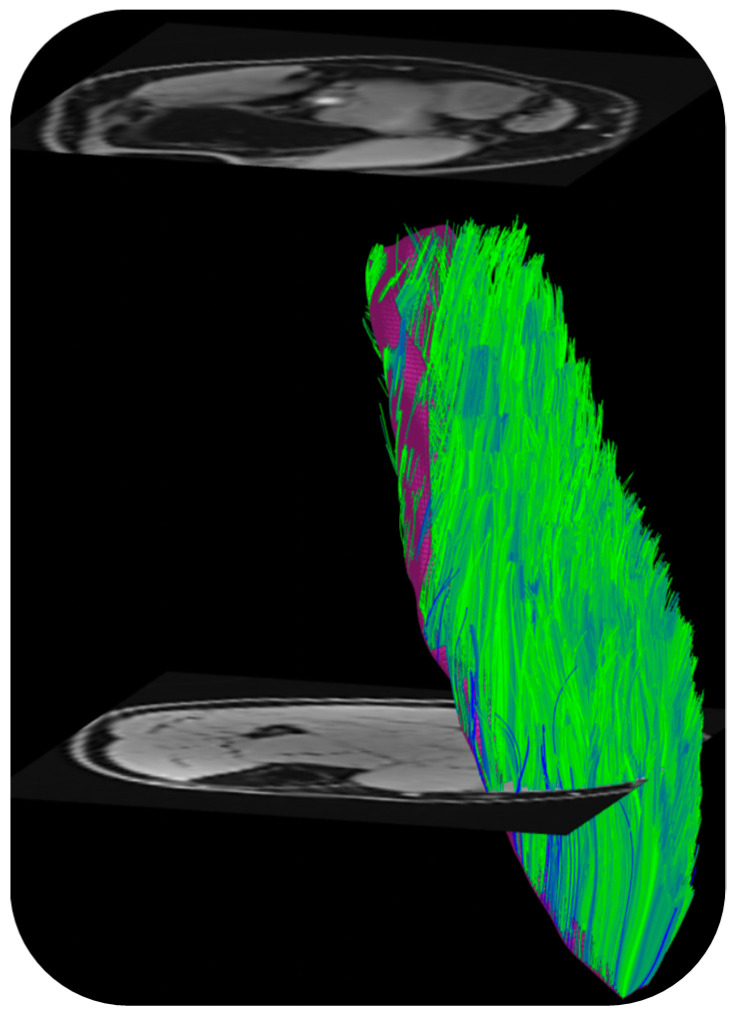
Fibers tracked from the deep aponeurosis (aponeurosis surface shown in deep purple mesh seen behind the muscle fibers in green) of the medial gastrocnemius using the MATLAB toolbox in Ref. [58]. (unpublished work, Master’s research work by Ngara Bird in the authors’ lab).

DTI also provides the basis of fiber tracking: the direction of the primary eigenvalue is extracted from the computed tensor so that the ‘fiber’ direction is available at each voxel [57]. Fiber tracking algorithms use the primary eigenvector direction for 3D muscle fiber tractography. The tracking starts from either a manually or automatically identified region of interest and terminated when stopping criteria based on FA range, max angular change per tracking step, and/or anatomical boundary are met. There are several freeware programs that were developed originally for brain imaging that can be adapted for muscle DTI and fiber tractography as well [57]. Recently, a DTI Matlab toolbox was released that allows users to perform tractography as well as to obtain muscle architectural parameters including fiber length, pennation angle, and curvature [58]. Figure 3 is an example of fiber tracking in the medial gastrocnemius using this toolbox customized to the acquisition in the authors’ lab. 

In order to understand the changes in DTI indices with conditions such as disease, exercise, or disuse, it is important to know the factors that affect diffusion. While the resolution of DT-MRI precludes direct observations at the tissue microscopic scale, the DTI indices may allow for indirect inferences about the microarchitecture of skeletal muscles. The measured diffusion indices reflect both intracellular and extracellular water volumes and a change in either (cell swelling and/or extracellular edema) will result in changes in the diffusion eigenvalues [56]. Other potential influences on the diffusion properties of muscle include changes in cell diameter and membrane permeability changes [59]. While there is general consensus that the direction of the lead eigenvector corresponds to the muscle fiber direction, there is less certainty about the two eigenvectors corresponding to the secondary and tertiary eigenvalues, respectively. Galban et al. proposed that the second eigenvalue, λ2, corresponds to diffusion in the endomysium while the third eigenvalue, λ3, reflects intracellular diffusion and is thus sensitive muscle fiber diameter [60]. Karampinos et al. proposed an interesting diffusion tensor model that considers the cross-sectional asymmetry of muscle fiber geometry [61]. In the latter model, diffusion occurs within the muscle fiber and the extracellular space and λ2 and λ3 reflect the principal diameters of the elliptical cross-sectional area of the myofibrils. Recent diffusion modeling studies support the model by Karampinos et al. where reductions in asymmetry of fiber morphology are seen in the case of disuse simulated by unilateral limb suspension and in a cross-sectional study of aging effects [62,63]. It is potentially likely that changes in fiber diameter would be reflected in changes in one or both of λ2 and λ3 and in FA. In summary, diffusion indices are related in a complex manner to free water in the different compartments, cell wall permeability, as well as muscle fiber diameter and cross-sectional asymmetry.

The application of DTI to characterize disease conditions is detailed later while a brief summary of studies on normal subjects is provided here. DTI-derived indices have been shown to be sensitive to age [60,64], and environmental factors (disuse, exercise) [61,64,65]. It should be noted that diffusion is strongly temperature dependent (temperature coefficient: ~2.4%/°C). For in vivo imaging, the temperature dependence may be important in diffusion imaging performed post-exercise [66]. Some of the observed increase in diffusion post-exercise can be attributed to an increase in temperature [67]. Age-related effects of DTI changes in the calf plantarflexors have been attributed to muscle atrophy or to the combined effects of an increase in extracellular volume and a decrease in muscle fiber diameter (from muscle atrophy) [60,64]. Froeling et al. reported that eigenvalues and FA were increased in thigh muscles of amateur long-distance runners up to 2 days after running a marathon [65]. The combined application of DTI and T2 mapping allowed the differentiation of microstructural changes caused by active exercise or endurance training [68]. Malis et al. found that all eigenvalues decreased with disuse simulated by unilateral limb suspension and diffusion modeling yielded smaller diameter and more symmetric fibers post-suspension [62].

In addition to the information provided by the DTI-derived indices, DTI also enables the study of tissue architecture through the ability to perform fiber tractography [69]. Fiber tracking in calf, thigh, and forearm muscles, reproducibility, and validation of the architectural parameters have been reported [70]. Furthermore, a multi-center trial including six MRI 3T sites and five traveling subjects reported excellent reproducibility of DTI and architecture measures in calf muscle with semi-automated segmentation of the calf muscles [70]. DTI fiber tractography has also been performed outside of the extremity muscles; in the masseter muscle fiber tracking confirmed regional differences in the fiber orientation change between different mandibular positions [71]. Fiber tractography has also enabled 3D visualization of the three major levator ani subdivisions, which can inform in vivo functional anatomy [72]. Interest in the DTI of pelvic floor muscles was triggered by initial results that showed fiber tractography might be able to reveal microstructural abnormalities in the pelvic support that are not noticeable using conventional MRI techniques [73]. DTI-based fiber tracking also identified age-related significant differences in fiber length and pennation angle of the gastrocnemius muscles between young and senior subjects; these results agreed with ultrasound measurements [64].

**Figure 4:**
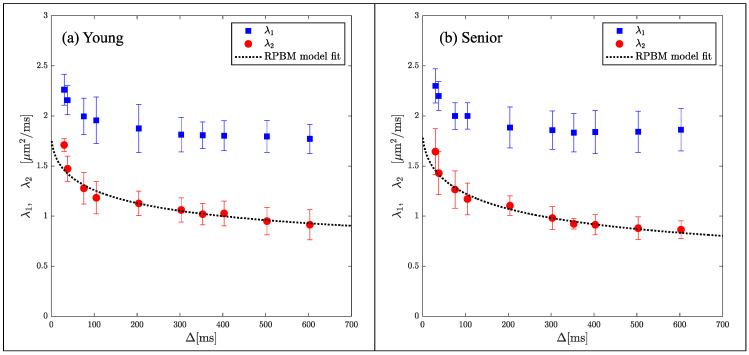
Average RPBM model fits of λ_2_(t) for the groups of young (**a**) and senior (**b**) participants, respectively. The points are experimentally determined while the dashed line is the model-derived fit to the eigenvalue. Reproduced with permission from the authors in Ref. [63].

While indices derived from DTI are sensitive to tissue microstructure, they are not direct measures of tissue microstructure. Models of diffusion in muscle have been proposed that are customized to the geometry and tissue subtypes in skeletal muscle. The Random Permeable Barrier Model (RPBM) has been applied to normal muscle, to monitor the effect of exercise on muscle tissue microstructure in normal and diseased conditions as well as to tracking induced atrophy and recovery (Reference [74] and references within). The RPBM model treats muscle as a volume with randomly oriented infinite flat semipermeable membranes and the time dependence of the transverse diffusion coefficient is fit to the model to extract parameters of the tissue microstructure. The RPBM study of atrophy found that the myofiber diameter was a stronger predictor of atrophy than either anatomical measurements such as cross-sectional area or empirical diffusion parameters [74]. The RPBM applied to a cross-sectional study of young and senior subjects revealed that fiber diameter from RPBM fits compared to that from histology had the highest correlation for the fit to λ_2_(*t*); these fits also predicted a decrease in fiber diameter and an increase in cell permeability with age (Figure 4) [63]. The age-related patterns in λ_2_(*t*) and λ_3_(*t*) could tentatively be explained from RPBM fits; these patterns may potentially arise from a decrease in fiber asymmetry and an increase in permeability with age [63]. DT-MRI RPBM metrics have recently been shown to agree with histology in Becker’s dystrophy including muscle fiber size and variability indicating that the modeling approach shows promise as imaging biomarkers for muscular dystrophies [75]. In addition to modeling the diffusion data to extract microarchitectural data, a complementary approach is to perform ex vivo high-resolution imaging at high field strengths to evaluate the diffusion characteristics in the different compartments of the muscle (e.g., fascicle and perimysium). For example, high-field imaging at 9.4T of the peripheral nerve has enabled the depiction of the anisotropic diffusion within the fascicles and perineurium [76] It should be noted that while there are clearly SNR advantages for high-resolution imaging at high fields, disadvantages include image artifacts due to inhomogeneity in the main magnetic field and radiofrequency field, as well as errors in chemical shift localization. 

### 2.5. Fibrosis Quantification

Most of the MRI quantification methods to document compositional changes with pathology have focused on quantification of fat fraction. However, it should be recognized that another major change that occurs in skeletal muscle is fibrosis, i.e., the replacement of contractile tissue by connective tissue that has a high percentage of collagen [77]. The replacement of contractile tissue in fibrosis has a greater negative impact than fat infiltration since the latter only affects the amount of muscle tissue while the former affects both the contractile tissue volume as well as the ability to transmit force [78]. In aging muscle, the loss of muscle mass is disproportionately smaller than the loss of muscle force [79]. Some of the force loss has been predicted from computational modeling to arise from impairment in lateral transmission of force caused by an increase in the connective tissue (increase in width of the extracellular matrix) [80]; this was also indirectly inferred from dynamic studies of muscle function [81,82]. Fibrosis is also present in muscular dystrophies such as Duchenne Muscular Dystrophy (DMD) and importantly, an increase in endomysial tissue occurs before any degeneration in skeletal muscles can be detected [78]. Recognizing the contribution of fibrosis to DMD, anti-fibrotic therapies have been developed [77]. Fibrosis is also present in metabolic myopathies [83], scleroderma [84], and in ALS [85], and contributes to disease progression. Several therapeutic approaches targeting pro-fibrotic pathways are under development [84]. However, unlike MRI studies quantifying fat infiltration, there are very few MR imaging studies quantifying fibrosis in these pathologies. A recent MRI study of extracellular volume (ECV) fraction in peripheral muscle of systemic sclerosis patients suggests diffuse fibrosis and an association of ECV with suspected myopathies [86]; this latter study underlines the importance of MR imaging of fibrosis to potentially characterize muscle involvement and response to treatment.

The above studies show that MRI techniques to characterize fibrosis and monitor response to therapy will be a very useful tool for evaluation of neuromuscular diseases. Unfortunately, there are no established MRI approaches to directly image fibrosis as there are for quantification of fatty infiltration [8,9]. Here, we discuss two techniques (magnetization transfer contrast and ultralow TEs) that have not yet been fully established but show promise as imaging markers of fibrosis. Collagen and other macromolecules of the extracellular matrix as well as their hydration water molecules have very short T2s such that they are not ‘visible’ on conventional images acquired with a TE of 5–10 ms. However, these very short T2 species can be imaged indirectly via magnetization transfer contrast or by imaging at extremely low TEs to capture the signal from even the very fast-decaying protons.

It should be noted that fat and fibrosis quantification will be affected by several factors: pulse sequence, image analysis to extract indices of fat and fibrosis, as well as manual/semi-manual segmentation of the muscle or in the placement of regions of interest (ROIs). Fat quantification is in a more advanced state of development than fibrosis quantification. The accuracy of fat quantification across multiple vendors, field strengths, and pulse sequences has been established [38]. Along the same lines, it is also important to develop fibrosis phantoms for validation. The fat and/or fibrosis percentage is then extracted from the parametric images either for the whole muscle or in user-selected ROIs. If the whole muscle segmentation and ROI analysis have manual components, it is important to define specific criteria for the manual interventions as well as to ensure consistency of these criteria across the control cohorts and those with pathology. It is also be important to conduct inter- and intra-user studies to establish the reproducibility of qMRI. 

#### 2.5.1. Magnetization Transfer Contrast

Magnetization Transfer (MT) describes the interaction of tissue water protons that reside in different environments, encompassing the “free” water proton pool responsible for the conventional MR imaging signal intensity and the “restricted” proton pool where protons are bound to macromolecules [87]. Protons in the bound pool, such as those bound to myelin, collagen, and proteoglycan, have a very short T_2_, making it difficult to image them directly [87]. However, a selective off-resonance radio frequency (RF) pulse can be applied such that the free pool remains unperturbed, while protons in the bound pool are saturated. The exchange between the excited (saturated) bound pool and the free pool effectively reduces the free pool net magnetization. Skeletal muscle exhibits a strong magnetization transfer contrast (MTC) though the origin of this contrast is still not definitively established. The primary contribution is hypothesized to come from the collagenous proteins of the extracellular matrix [88,89], but there is increasing evidence that there are contributions from the large abundance of contractile proteins [90]. 

The simplest imaging technique to obtain an estimate of the MT effect is the magnetization transfer ratio (MTR) calculated from the signal intensity with and without the off-resonance RF pulse. Since it requires only two measurements, it is fast and clinically practical [87]. However, MTR values are pulse sequence, T1, and RF field homogeneity dependent [87]. On the other end of the spectrum, the quantitative magnetization transfer (qMT) techniques fit appropriately acquired MRI data to a two-pool model of magnetization exchange between protons bound to macromolecules and free protons, providing estimates of the relaxation and exchange rates as well as the ratio of the sizes of these two pools [88,89]. A faster, computationally simple, semi-quantitative index of Magnetization Transfer that does not fit to a two-pool model but derives an index of Magnetization Transfer denoted as MT_sat_ has also been implemented [91]. This index, unlike MTR, is independent of pulse sequence, T_1_ and RF field homogeneity. MTR, qMT, and MT_sat_ mapping have been reported for skeletal muscle [88,89,92,93,94]. 

Age- and gender-based differences in MTR (corrected for B1 inhomogeneities) and MT_sat_ have been reported [92,93,94]. MTR and MTsat were both correlated negatively with age. It should be noted that of three quantitative markers (T2, fat fraction, and MTR), T2 and fat fraction were significantly positively corelated while MTR (adjusted for fat fraction as a covariate) was significantly negatively correlated with age [95]. However, in terms of effect size, MTR was the largest indicating that this metric may be a clinically useful biomarker. MTsat (with fat suppression), like MTR, was also significantly negatively correlated with age and was higher in males than females [93,94]. These results are contradictory to the hypothesis that the MT effect in muscle is a measure of the collagen macromolecule. If that hypothesis is correct, then a positive correlation of MT indices with age is anticipated since fibrosis (and thus, collagen) increases with age. Morrow et al. concluded that age-related decrease in MTR may arise from myofiber quality and density changes with age [92]. Support for the contribution of contractile proteins to MTR also comes from a rat model study of MTR to track muscle fiber formation after injection of human muscle progenitor cells for development of muscle tissue [90]. In the latter study, MTR increased with myogenesis and correlated well with muscle contractility measurements. These studies suggest that biopsy studies are critical to show the correlations of MT indices to macromolecules in muscle. 

#### 2.5.2. Ultralow TE (UTE) Imaging

**Figure 5:**
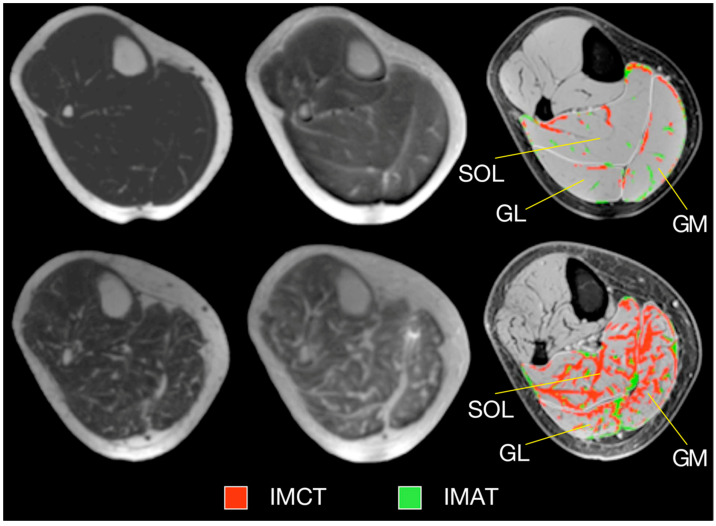
Typical examples of MR images and resulting tissue segmentation in young and older women. Left: Water-saturated FGRE (showing IMAT), Middle: UTE (showing IMCT), Right: Standard morphological images with superimposed outer contours of muscles and the result of the automated tissue segmentation. Images in top and bottom row represent one young and one old subject, respectively. (SOL—soleus muscle, GM—medial gastrocnemius muscle, GL—lateral gastrocnemius muscle, IMAT—intramuscular adipose tissue, IMCT—intramuscular connective tissue.) Reproduced with permission from the authors in Ref. [41].

Ultralow TE imaging, as the name implies, acquires the signal at TE values as low as 8 μs; typically sequences with TEs in the range of 8 μs to 200 μs are classified as UTE imaging. Imaging at 8 μs–200 μs will render many short T2 species visible. Figure 5 shows fibrotic and adipose voxels (after thresholding) extracted from the calf plantarflexors using a combination of UTE (for low T2 tissues) and IDEAL (for fat) imaging in a cross-sectional study of young and elderly subjects [41]. The latter study showed significant increase in fat and connective tissue fraction in the older cohort. 

One of the big challenges in extracting the short T2 species is that signal from the long T2 species is overwhelming. One of the methods suggested is to subtract a longer TE image from a UTE image (there is no contribution from short T2 species in the longer TE image); however, the image subtraction is very sensitive to magnetic susceptibility effects resulting from the long T2* weighting of the images and the initial fast dephasing of the multiple fat resonances mimics short T2 tissue and thus their signal is not subtracted. To overcome this, Araujo et al. [95] suggested an extension of the dual-echo method that considers the T2* decay of long T2 components and also corrects for the oscillating behavior of the signal from the different lipid resonances in fat. This idea was also implemented in another study that integrated the fat fraction and T2 information from an IDEAL sequence with a dual-echo UTEs sequence to extract macromolecular fractions (MMF) [96]. The latter study extracted MMF from UTE images acquired at 30 μs and at 200 μs, illustrating the potential to identify different macromolecules in muscle (e.g., collagen, contractile proteins) by selection of the appropriate TE for the UTE echo. 

### 2.6. Strain and Strain Rate Imaging

Strain and strain rate are kinematic properties that can be derived from the displacement (strain)- and velocity (strain and strain rate)-encoded magnetic resonance (MR) images and have been used to characterize deformation in skeletal muscle [78,81,82]. Strain describes how the tissue is deformed with respect to a reference state and requires tissue tracking. Strain rate describes the rate of regional deformation and does not require tracking or a reference state since it is an instantaneous measure. A positive strain or strain rate indicates a local expansion whereas a negative strain or strain rate indicates a local contraction. A number of dynamic studies have used velocity-encoded phase-contrast (VE-PC) sequences to extract muscle tissue velocities during a contraction paradigm. Other sequences like DENSE encode displacement while MR tagging is an alternate sequence where the tagged lines/grid are tracked to quantify strain [97,98]. 

**Figure 6:**
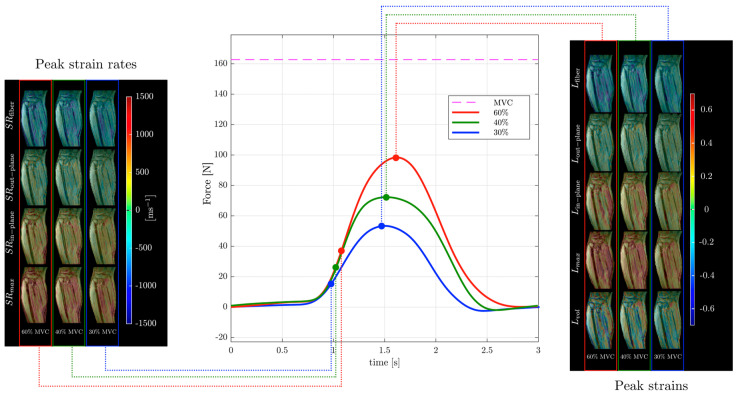
Temporal variation in forces exerted by a young subject averaged during the MR data acquisition for different force levels (center panel) along with corresponding strain (right panel) and strain rate (left panel) colormaps at the peak values of strain or strain rate along the fiber during the contraction phase of the dynamic cycle for 60% (left column), 40% (middle column), and 30% MVC (right column). The colormap bars are shown in each panel. The temporal frames at which the peaks during contraction occurred for strain and strain rate are marked on the force curves. While the peak in strains occurs at the maximum force reached, peak in strain rates occurs earlier and roughly corresponds to the maximum slope of the force–time curve in the contraction cycle. Reproduced with permission from the authors in Ref. [99].

Strain and strain rate tensor imaging of the lower leg was used to study age-related differences between younger and older subjects [82,99]. Maximum shear strain was shown to correlate with force in this cohort of young and old subjects [99]. Figure 6 shows images of different indices extracted from the strain and strain rate tensor data of the lower leg during isometric contraction at different %MVCs of a young subject from Reference [99]. Strain rate tensor imaging of disuse atrophy also identified maximum shear strain as a significant predictor of force loss with disuse [78]. The authors of the latter paper speculated that the dependence of force on shear strain may be related to the mechanical properties of the extracellular matrix that may become stiffer with age [81,99]. Recent developments in accelerated VE-PC imaging using compressed sensing have enabled multi-slice imaging and extraction of the 3D strain tensors [99]. 

## 3. In Vivo Clinical Applications

### 3.1. Duchenne Muscular Dystrophy (DMD)

DMD is an X-linked recessive genetic disease caused by mutation of the dystrophin gene and is characterized by severe, progressive muscle wasting. The dystrophin protein connects the muscle cytoskeleton with the extracellular matrix and prevents the muscle membrane from being damaged during muscle contraction [100]. Therefore, loss of the dystrophin protein leads to degeneration of muscle fibers, chronic inflammation, progressive fibrosis, and muscle replacement by fat. While currently there is no cure for DMD, there are many new treatments that show promise; some of these treatments are now in clinical trials [101]. Furthermore, there are rehabilitation training programs to improve muscle function [102]; this training has been shown to be most effective in affected muscles in the early stages of the disease [102]. Baseline and longitudinal assessment of subjects with DMD can be realized by sensitive non-invasive biomarkers. These biomarkers should be able to objectively characterize disease severity and progression in muscles as well as the response to pharmacological and/or rehabilitation treatment. MRI enables non-invasive, repeatable, and objective assessment of individual muscles. It is also evident from Section 2 on the techniques that the consequences of the loss of dystrophin protein listed above can be tracked using MRI. A recent meta-analysis of publications of MRI in DMD till 2019 concluded that additional larger clinical trials, more validation studies to histology standards, and multiparametric MRI mapping are needed to establish MRI as a biomarker in DMD [103]. 

**Figure 7:**
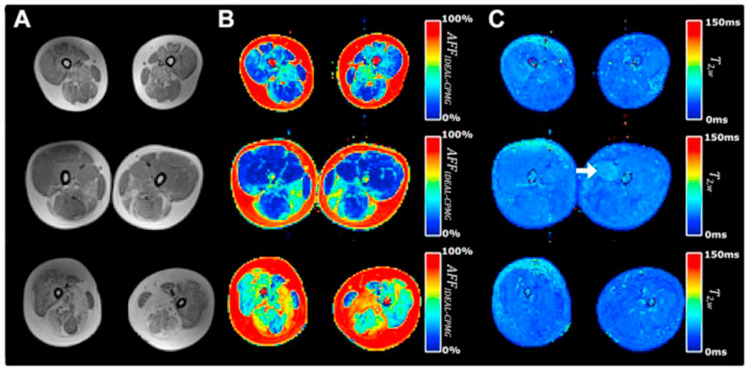
Representative T1-weighted and IDEAL-CPMG images of the thigh muscles in three subjects with DMD. A T1-weighted image (**A**), T2-corrected fat fraction map (**B**), and water T2 map (**C**) are shown representing subject anatomy, changes in muscle apparent fat fraction (AFFIDEAL-CPMG), and muscle water T2 (T2w IDEAL-CPMG), respectively, in the thigh muscles of subjects with DMD. Different severity of fatty degeneration is present in the thigh muscles of each subject, whereas inflammatory activity is sparse and seen in only few muscles (arrow). Reproduced with permission from the authors in Ref. [107].

There are many clinical studies that have established qMRI as being able to successfully characterize and to monitor DMD. Confirming earlier work, Yin et al. showed the T2 of thigh muscles of DMD subjects was significantly longer than control subjects and that functional outcomes were significantly correlated with the overall mean T2 relaxation time [48]. The earlier papers focused on quantifying fat infiltration and used T2 as a surrogate marker of fat and confirmed that fat fraction was highly positively correlated with fat fraction from MR spectroscopy [104]. Kim et al. explored fat-suppressed T2 mapping for edema quantification and concluded that fat fraction rather than edema was more highly correlated with clinical evaluations [105]. The calf muscles have also been studied as there is slower progression in the distal muscles allowing extended longitudinal monitoring [106]. This latter study found significant correlations between the change in all soleus T_2_ (nonfat suppressed T2) and change in functional measures over two years. Mankodi et al. implemented IDEAL-CPMG to extract fat fraction and T_2,w_ in the thigh muscles of subjects with DMD and healthy controls and concluded that fat fraction and T_2,w_ may be useful as independent biomarkers of fat infiltration and inflammation, respectively [107]. Figure 7 shows that IDEAL-CPMG can disambiguate fat infiltration from inflammation in the fat fraction and water T2 maps. A longitudinal study of DMD subjects over a one-year time period used quantitative MRI (three-point Dixon for F/W, T2, and T1 mapping) to identify the most responsive muscle and predict subclinical disease progression in functionally stable patients. The latter study concluded that qMRI biomarkers are responsive to disease progression, can also detect subclinical disease progression, and that the gluteus maximus is the most responsive to disease progression [108]. 

The majority of quantitative MRI studies on subjects with DMD have focused on fat fraction and T2 mapping. However, DTI has also been used to identify differences in fiber organization in diseased and healthy muscle tissue. Hoojimans et al. combined DTI with quantitative in vivo measures of mean water T2, %fat, and SNR to evaluate their effects on DTI parameter estimation in DMD subjects and healthy controls [109]. Analyzing voxels with a baseline SNR above a certain threshold (to exclude voxels with high fat fraction), the latter study reported significantly greater values for MD in the tibialis anterior ((1.78 ± 0.04 (DMD), 1.61 ± 0.04 (control), *p* < 0.009) and for the third eigenvalue in the anterior tibialis (1.33 ± 0.03 (DMD), 1.17 ± 0.03 (control), *p* < 0.007), and in the lateral gastrocnemius muscles (1.31 ± 0.023 (DMD), 1.15 ± 0.02 (control), *p* < 0.001), and no significant change is fractional anisotropy in DMD subjects compared to controls. This study underlines the need to account for the effect of confounders on diffusion indices to detect true between-group differences between controls and subjects with DMD [109]. The authors concluded that the increased MD and the third eigenvalue in the TA muscle most likely reflect the pathophysiology in subjects with DMD [110]. Another study of DTI of thigh muscles of DMD subjects and healthy controls showed that, for all the thigh muscles, the MD was higher and FA values lower compared to healthy controls and correlated with grade of fatty infiltration; these findings indicate that DTI can be used to characterize DMD-induced muscle damage and extent of disease severity [110]. More DTI studies with particular attention to effective fat suppression and the baseline SNR of analyzed voxels are required to obtain consistent and reliable measurements independent of the degree of fat infiltration.

### 3.2. Idiopathic Inflammatory Myopathies (IIM)

The idiopathic inflammatory myopathies (IIMs) are a group of autoimmune conditions characterized by inflammation of muscle (myositis) that presents with weakness, elevated muscle enzymes, inflammatory infiltrates on biopsy, and can be accompanied by other systemic manifestations [111]. It results in inflammation in other organ systems, resulting in widespread organ dysfunction, increased morbidity, and early mortality. The IIMs include dermatomyositis (DM), necrotizing autoimmune myopathy (NAM), sporadic inclusion body myositis (sIBM), overlap myositis and antisynthetase syndrome (ASyS), and polymyositis (PM) [111]. Qualitative and quantitative MRI play an important role in IIM not only as a diagnostic tool but also in monitoring progression and response to therapy [112]. 

Myositis is accompanied by both fatty infiltration and inflammatory changes [111]. Qualitatively, fatty infiltration is seen as hyperintensity on T1-weighted images while the fat fraction can be quantified by a three-point Dixon or more accurately by sequences such as IDEAL or its equivalents [35,36]. Qualitative detection of inflammatory changes is performed on T2-weighted sequences where they appear as hyperintensities. It is important to note that fat should be suppressed on T2-weighted sequences since it also presents as a hyperintense signal [112]. T2 mapping is used for quantification of inflammation, and as in T2-weighted imaging, it is important to suppress fat to exclude the contributions from fat infiltration that accompany chronic muscle damage. Yao et al. showed the feasibility of generating fat-corrected T2 maps by incorporating information from fat fraction maps; they show that T2 was as responsive as fat-corrected T2 when either is used for qualitative scoring [45]. It should also be noted that T2 values can be as high as 50 ms (15 ms above normal condition) in untreated IIM, values that are rarely seen in other muscle conditions [111]. Another important aspect is that in the IBM type of IIM, T_2,w_ showed early changes before significant intramuscular fat accumulation, providing potential measures of early disease before irreversible changes occur [106]. The anatomy covered in IIM is the lower extremity and sometimes restricted to only the thighs but whole-body imaging can be useful to detect patterns of muscle involvement and fatty infiltration specific to each IIM [111,112].

Diffusion tensor imaging has been applied to study thigh muscles of subjects with myositis (specifically PM and DM) [113,114]. Wang et al. found that ADC of edematous muscle was significantly increased compared to normal control subjects as well as to non-edematous muscle in the vastus medialis (2.01 ± 0.03 (PM/DM), 1.72 ± 0.08 (control), *p* < 0.017); vastus intermedius (2.07 ± 0.21 (PM/DM), 1.70 ± 0.11 (control), *p* < 0.017); adductor magnus (2.03 ± 0.16(PM/DM), 1.71 ± 0.13 (control), *p* < 0.017); and semimembranous (1.98 ± 0.23(PM/DM), 1.64 ± 0.14 (control), *p* < 0.017) (ADC values in μm^2^/ms) [114]. This latter study also found that significant decrease in FA in edematous muscle of subjects was found compared to normal control subjects in the vastus medialis (0.22 ± 0.03 (PM/DM), 0.30 ± 0.06 (control), *p* < 0.017); vastus intermedius (0.22 ± 0.03 (PM/DM), 0.30 ± 0.06 (control), *p* < 0.017); adductor magnus (0.19 ± 0.04 (PM/DM), 0.25 ± 0.03 (control), *p* < 0.017); and semimembranous (0.23 ± 0.04 (PM/DM), 0.29 ± 0.04 (control), *p* < 0.017). This is not surprising since inflammation increases free water (seen as an increase in T_2,w_) and DTI indices may be tracking the changes in free water. The role of diffusion tensor imaging in myositis awaits further studies.

### 3.3. Pompe Disease

**Figure 8:**
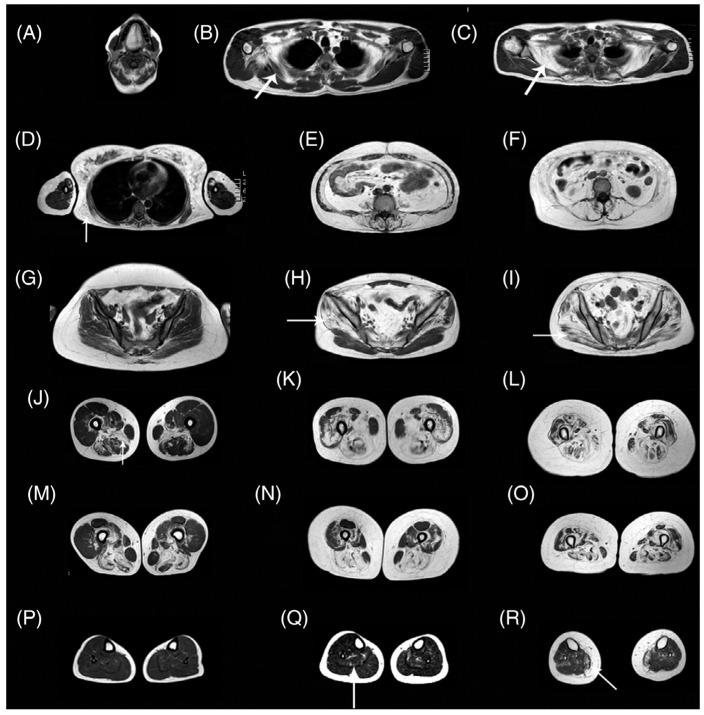
Whole-body T1w imaging of patients with late-onset Pompe disease. (**A**) Involvement of tongue is observed. In the scapular girdle, the subscapularis (arrow in **B**,**C**) and latissimus dorsi (arrow in **D**) are affected, yet the deltoid, biceps, and triceps are not typically involved. Paraspinal and abdominal muscles are typically affected (**E**,**F**). The gluteus minimus and medius (arrow in **H**) are affected earlier than the gluteus maximus (arrow in **I**). Patients in the early stages of disease may have no glutei involvement (**G**). In the thigh, the adductor magnus and long head of biceps are involved earlier (**J**,**M**), whereas posterior muscles and the vasti are affected later in the progression (**K**,**N**). Eventually, all muscles of the thigh are affected (**L**,**O**). A proximal-to-distal gradient in the vasti is usually identified (**J**–**M**,**K**–**N**), although it is lost in advanced stages (**L**–**O**). Lower legs are usually spared (**P**), although mild replacement of the soleus (arrow in **Q**) and media gastrocnemius (arrow in **R**) can be observed. The images shown are from seven patients. T1w, T1-weighted. Reproduced with permission from the authors in Ref. [115].

Pompe disease is characterized by a deficiency of acid alpha-glucosidase (AAG) that results in muscle weakness and a variable degree of disability [115]. AAG deficiency leads to accumulation of glycogen within the lysosomes of the cells in multiple tissues, including skeletal, cardiac, and smooth muscle. There is an approved therapy based on enzymatic replacement (ERT) alglucosidase alfa that has modified disease progression [116]. qMRI can potentially detect subtle changes with treatment in Pompe disease in muscle structure, fat, and glycogen content even before the effects are seen clinically in muscle function tests [115]. An excellent review of MRI in Pompe disease is available in Reference [115]. Figure 8 shows whole-body T1-weighted MRI revealing typical patterns of muscle involvement in late-onset Pompe disease (LOPD). 

Rehmann et al. used qMRI including quantitative Dixon for fat fraction and diffusion tensor imaging to image the thigh muscles of subjects with LOPD and compared to healthy controls. The DTI metrics included mean diffusivity (MD), eigenvalues (λ_1–3_), radial diffusivity (RD), and fractional anisotropy (FA) [117]. They found that even thigh muscles with <10% fat fraction showed significant differences in all the diffusion parameters except for FA; all the diffusion values were significantly lower and this has been hypothesized to arise from the accumulation of glycogen in muscle fibers that restricts water mobility and therefore, DTI could potentially reveal important structural changes early in the progression of the disease even prior to fatty degeneration [117]. The EMBASSY study followed 16 LOPD subjects on ERT and assessed the changes from baseline to 6 months using histology-based (% tissue area of glycogen), MR imaging (T1w, T2, fat fraction), and muscle function biomarkers. The glycogen area decreased and function improved but there were no changes in the MR assessment over the 6-month period [118].

Long-term follow-up of LOPD subjects treated by ERT for fat infiltration in psoas and paraspinal muscles based on conventional MRI revealed significant increase between baseline and at 39 months which also correlated with a decrease in performance [119]. However, both fat fraction and performance did not change in the long-term follow-up (63 months), showing promise for ERT [118]. A follow-up of LOPD subjects with qMRI showed that fat fraction increased significantly in every thigh muscle by an average of 1.9% per year in ERT-treated patients, compared with 0.8% in pre-symptomatic patients [120]. The authors of the latter study also observed a significant correlation between changes in fat fraction and changes in muscle function tests; this potentially indicates that fat fraction and muscle function tests can be considered good outcome measures for clinical trials in LOPD patients [120]. These studies show that future research with larger cohort size and long-term follow-up of LOPD subjects with ERT are required to determine the efficacy of the treatment. qMRI will be clearly very important as newer treatments are introduced and long-term follow-up is needed to assess disease status. 

### 3.4. Sarcopenia

Sarcopenia is the progressive loss of muscle mass and strength that occurs with advancing age as well as with a number of long-term conditions [121]. It was originally defined by a loss of muscle mass but has been extended to skeletal muscle function with the latest definition from the European Group on Sarcopenia in Older People (EWGSOP): “a muscle disease rooted in adverse muscle changes that accrue across a lifetime” [121]. 

A recent review performs a comprehensive survey of all studies that reported MRI-derived biomarkers related to sarcopenia [122]. This review reveals that the primary anatomical regions imaged were the thigh followed by the trunk. Currently, MRI allows the assessment of muscle quantity and quality (MQQ) using T1w, T2w for cross-sectional area measurements, inflammation/edema from T2w mapping, proton density fat fraction, and fat free muscle mass from Dixon or variant sequences, extramyocellular and intramyocellular lipid fractions from Magnetic Resonance Spectroscopy, ADC, FA, and fiber architecture (length and pennation angle) from DTI [41,64,123,124,125,126]. Yang et al. have shown using a modified Dixon sequence that muscle CSA and intermuscular fat area at the 50% femur length highly correlated with muscle and intermuscular fat volumes estimated from the middle third of the thigh in a cohort of older subjects classified as normal, obese, sarcopenia, and sarcopenia-obese [124]. 

An MR compositional study established that aging causes significant changes in skeletal muscle composition, with marked increases in non-contractile tissues (adipose and fibrosis infiltration) [41]. Such quantification of the remodeling process is likely to be of functional and clinical importance in elucidating the causes of the disproportionate age-associated decrease in force compared to that of muscle volume. Melville et al. imaged the quadriceps musculature of young healthy females and compared them to non-frail and pre-frail/frail older females [125]. MR imaging assessment included diffusion tensor imaging, T2 mapping, and quantitative fat fraction using MRS. The latter study found that pre-frail/frail adults demonstrated increased FA compared to young controls and non-frail adults with increasing T2 and intramuscular fat among the control, non-frail, and pre-frail/frail categories [125]. Another cross-sectional DTI study of young and senior (non-frail) subjects showed significantly higher eigenvalues and trended to a higher FA and significantly shorter fiber lengths and smaller pennation angles in the gastrocnemius muscles of the senior cohort compared to the young cohort [64]. Cameron et. al. extracted DTI indices (fractional anisotropy and mean diffusivity) and architecture (fiber length, pennation angle, PCSA) in thigh muscles in a cohort of 94 subjects with an age range 22–89 years [126]. The latter study showed skeletal muscle architectural changes with aging and intermuscular differences in the microstructure. 

Though MRI has a number of quantitative assessments of muscle quality and quantity, these remain in the realm of research in sarcopenia due to the lack of imaging and analysis standardization, complex post-processing, and long scan times. More studies focused on validation as well as on the identification of simpler MR metrics (acquisition and/or processing) will serve to expedite establishment of MRI as an imaging biomarker of sarcopenia. Large-scale multi-parametric MR imaging studies on cohorts comparing heathy young, active older, pre-frail, and frail older subjects will be required to determine thresholds for each MR metric for the three sub-groups of older subjects to establish MRI-based biomarkers of sarcopenia.

### 3.5. Muscle Injury

MRI is routinely used to assess the severity in sports-related muscle injuries, and combined with clinical evaluation, used to predict ‘return to play (RTP)’ [127,128]. It is considered the reference standard for the evaluation of muscle injuries MRI aids in evaluating and in the management of sports-related muscle injuries. Furthermore, MRI can also evaluate the long-term changes following injury such as scarring and focus or diffuse fatty muscle atrophy [128].

**Figure 9:**
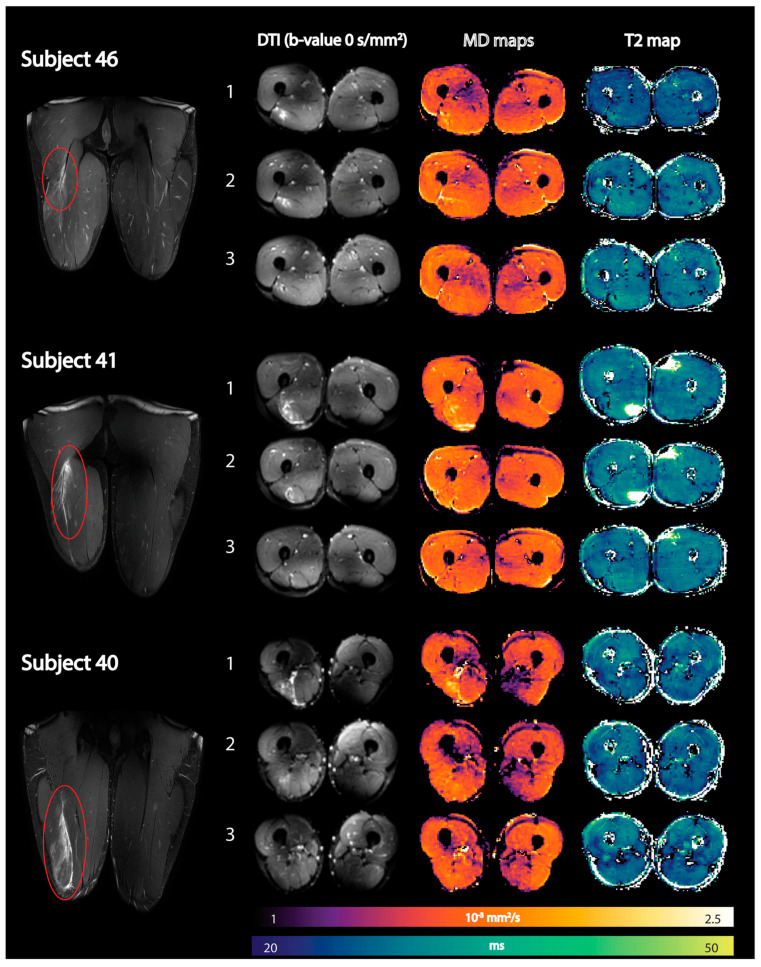
Representative images of three athletes showing coronal fat-suppressed T2-weighted images of the hamstring injury depicted by the red oval (first column) together with axial spin-echo EPI images (*b*-value = 0 s/mm^2^) (second column), reconstructed mean diffusivity (MD) maps (third column), and reconstructed qT2 maps (fourth column) at the three time points (time point 1: within 1 week postinjury; time point 2: 2 weeks after visit 1; and time point 3: at clinical return to play). DTI, diffusion tensor imaging; qT2, quantitative T2. Reproduced with permission from the authors in Ref. [130].

The integration of quantitative multiparametric MRI will increase the diagnostic efficiency and predictive power of MRI [128]. Most of the quantitative MRI studies thus far have focused on DTI and T2 metrics while some have evaluated the loss of muscle volume after injuries and in the rehabilitation period. Muhlenfeld et al. reported significant muscle volume loss (between 2% and 7%) in the upper thigh occurs in recreational soccer players assessed at three and at six weeks following a hamstring injury [129]. Diffusion tensor imaging (DTI) and T2 mapping have recently been applied to monitor recovery after an acute hamstring injury [130]. All DTI indices except FA were elevated compared to control muscles immediately after the injury and normalized during the recovery period. Mean T2 relaxation times in injured muscles were not significantly elevated compared with control muscles at any time point [130]. Figure 9 shows the baseline, mean diffusion, and T2 maps in three subjects at three time points after an acute hamstring injury. Future work should explore the potential of DTI indices to predict ‘return to play (RTP)’ and recovery times in athletes after an acute strain injury [127,128]. Biglands et al. also assessed the ability of T2 mapping, diffusion tensor imaging (DTI), and radiologists’ scores to detect muscle changes following acute muscle tear in athletes and to predict RTP [131]. While T2 and DTI measurements in muscle could detect changes due to healing following muscle tear, they were inferior predictors of RTP compared with the radiologists’ visual scoring. Bye et al. investigated mechanisms by which short-term resistance training (6 weeks) increases strength of partially paralyzed muscles in people with spinal cord injury (SCI) using DTI including fiber architecture and physiological cross-sectional area (PCSA) [132]. The lack of any change in muscle architecture post-training in this study suggests that short-term strength gains are due to increased neural drive or an increase in specific muscle tension [132].

While muscle biomarkers have been entirely devoted to markers of structure, composition, and fiber architecture, a few dynamic imaging studies have also been reported [97,98,99]. Silder et al. used velocity-encoded phase contrast imaging to map thigh muscle strains under active lengthening paradigms in subjects with prior hamstring injuries [133]. They found relatively larger localized tissue strains during active lengthening contractions near the proximal musculotendon junction from which they concluded that these large strains may predispose the proximal biceps femoris to injury. With faster and 3D imaging capabilities of the 4D-compressed sensing flow sequences, it is possible now to cover the entire thigh in the dynamic scan in 4–5 min [134]. This opens up exciting possibilities to establish imaging biomarkers of muscle function.

## 4. Conclusions

Quantitative MRI and imaging biomarkers are an active area of research and the multiparametric nature of MRI allows one to probe the muscle with different metrics. Some of these metrics have reached a stage of maturity to be granted the status of imaging biomarkers [10]. These mature biomarkers are morphological (volumes, cross-sectional areas), compositional (fat infiltration), and T2 mapping (inflammatory process, disease activity marker). The advent of deep learning methods is poised to make automated muscle segmentation a reality and with it, brings the ability to extract biomarker values in a consistent and accurate manner. These imaging biomarkers now need to be evaluated in large-scale clinical trials to determine their utility as outcome measures. Besides the mature muscle imaging biomarkers, there are other techniques that hold great promise and are in different stages of development: diffusion tensor imaging has already been shown to provide characterization of muscle that is distinct from the established biomarkers in normal and diseased states and fibrosis quantification which is still in its infancy. In addition, muscle proton and phosphorous MR spectroscopy also show considerable promise; these latter two topics are not covered here. Phosphorous spectroscopy of muscle was the subject of some of the earliest studies in biological samples and is a well-researched area that provides insight into energy metabolism, a metric not available through other MRI approaches. Proton spectroscopy is unique in its ability to quantify intramyocellular fat and also serves as a reference standard for quantifying adipose content. Other biomarkers of interest but not discussed here are MR elastography for muscle mechanical properties and MR perfusion for assessing blood supply to the skeletal muscle. As mentioned in the prior section, dynamic imaging of muscle opens up an unprecedented opportunity to identify a novel set of imaging biomarkers of muscle function. Along with technical advances in imaging sequences, image processing, and standardization, large-scale multi-institutional studies with well-defined outcomes measures in different disease states are required to advance and firmly establish qMRI in the arsenal of tools for the management of MSK disease conditions.
